# Distinct Functional Roles of β-Tubulin Isotypes in Microtubule Arrays of *Tetrahymena thermophila*, a Model Single-Celled Organism

**DOI:** 10.1371/journal.pone.0039694

**Published:** 2012-06-22

**Authors:** Sandra Pucciarelli, Patrizia Ballarini, Daniela Sparvoli, Sabrina Barchetta, Ting Yu, H. William Detrich, Cristina Miceli

**Affiliations:** 1 School of Biosciences and Biotechnology, University of Camerino, Camerino (MC), Italy; 2 Department of Earth and Environmental Sciences and Department of Biology, Northeastern University Marine Science Center, Nahant, Massachusetts, United States of America; University of Ottawa, Canada

## Abstract

**Background:**

The multi-tubulin hypothesis proposes that each tubulin isotype performs a unique role, or subset of roles, in the universe of microtubule function(s). To test this hypothesis, we are investigating the functions of the recently discovered, noncanonical β-like tubulins (BLTs) of the ciliate, *Tetrahymena thermophila*. *Tetrahymena* forms 17 distinct microtubular structures whose assembly had been thought to be based on single α- and β-isotypes. However, completion of the macronuclear genome sequence of *Tetrahymena* demonstrated that this ciliate possessed a β-tubulin multigene family: two synonymous genes (*BTU1* and *BTU2*) encode the canonical β-tubulin, BTU2, and six genes (*BLT1-6*) yield five divergent β-tubulin isotypes. In this report, we examine the structural features and functions of two of the BLTs (BLT1 and BLT4) and compare them to those of BTU2.

**Methodology/Principal Findings:**

With respect to BTU2, BLT1 and BLT4 had multiple sequence substitutions in their GTP-binding sites, in their interaction surfaces, and in their microtubule-targeting motifs, which together suggest that they have specialized functions. To assess the roles of these tubulins *in vivo*, we transformed *Tetrahymena* with expression vectors that direct the synthesis of GFP-tagged versions of the isotypes. We show that GFP-BLT1 and GFP-BLT4 were not detectable in somatic cilia and basal bodies, whereas GFP-BTU2 strongly labeled these structures. During cell division, GFP-BLT1 and GFP-BLT4, but not GFP-BTU2, were incorporated into the microtubule arrays of the macronucleus and into the mitotic apparatus of the micronucleus. GFP-BLT1 also participated in formation of the microtubules of the meiotic apparatus of the micronucleus during conjugation. Partitioning of the isotypes between nuclear and ciliary microtubules was confirmed biochemically.

**Conclusion/Significance:**

We conclude that *Tetrahymena* uses a family of distinct β-tubulin isotypes to construct subsets of functionally different microtubules, a result that provides strong support for the multi-tubulin hypothesis.

## Introduction

Microtubules are required for many fundamental processes of the eukaryotic cell, including mitosis and meiosis, intracellular translocation of organelles, maintenance of cellular architecture, and cellular motility. These cylindrical polymers are composed of αβ-tubulin heterodimers plus a variety of microtubule-associated proteins. In most eukaryotes, the α- and β-tubulins are encoded by small, multigene families, and each gene yields a distinct tubulin “isotype” [1], [2]. Although the tubulin isotypes of multicellular organisms were once proposed to be functionally equivalent [3], substantial evidence supports the multi-tubulin hypothesis – each tubulin isotype performs a subset of roles, whether highly specific or broadly generalized, in the universe of microtubule function(s) [4], [5]. Modulation of the levels of vertebrate class-III or -V β-tubulins, for example, has been shown to alter the dynamics and drug sensitivity of microtubules in cultured cell lines [6]–[9], and overexpression of βIII-tubulin is implicated in the resistance of tumors to tubulin-binding chemotherapeutics [10]–[12]. Furthermore, several congenital neurological disorders in humans result from mutations in distinct tubulin isotypes (reviewed by Tischfield and Engle [13]). In *Drosophila*, assembly of the sperm tail axoneme requires specific isotypes for formation of the central pair microtubules [14], [15] and for attachment of outer-arm dyneins [16]. Thus, specialized tubulin isotypes have evolved in the context of the stringent structural and functional constraints on microtubules [17].

The ciliated protozoan *Tetrahymena thermophila* has been used extensively as a model for studying microtubule-mediated cellular processes (reviewed by Gaertig [18]). This organism assembles and maintains within a single cell 17 distinct microtubular structures, a diversity that is comparable to that found collectively in the cells of multicellular organisms. Among the cytoskeletal structures formed by *Tetrahymena* tubulins and microtubule-associated proteins (MAPs) are basal bodies, cilia, and mitotic and meiotic spindles; other specialized, microtubule-based systems control cellular architecture, participate in physiological functions such as phagocytosis and osmoregulation, or are required for nuclear maturation [18], [19].

Formation and function of the microtubule systems of *Tetrahymena* is controlled by cell-cycle-dependent transcription of the nanochromosomes of its polyploid, somatic macronucleus, whereas the diploid, germline micronucleus is transcriptionally silent [20]. Prior to sequencing of its macronuclear genome, *Tetrahymena* was thought to possess a single *α-tubulin* gene and two synonymous *β-tubulin* genes, *BTU1* and *BTU2* [21], [22]. Given the apparently low diversity of tubulin genes in this and other ciliates, generation of a microtubule cytoskeleton with diverse functions has been attributed to specific posttranslational modifications of microtubules [19] and/or to the binding of specific MAPs [18].

The recent completion of the macronuclear genome sequence of *Tetrahymena* [23], [24] revealed three previously undescribed genes encoding α-like tubulins and six genes for β-like tubulins (abbreviated BLTs). This unanticipated observation immediately raised the possibility that diversity of tubulin isotypes in ciliates may contribute to the formation of functionally distinct subsets of microtubules. Thus, our objective is to determine by genetic manipulation whether the BLTs of *Tetrahymena* are functionally equivalent to, or different from, the canonical BTU1/BTU2 tubulins. The noncanonical BLTs are numbered from 1 to 6, but BLT4 and 5 are identical in protein sequence and are encoded by genes whose coding regions are also identical, consistent with recent gene duplication. (Hereafter, we will refer to this isotype as BLT4.) Each of the *BLT* genes is transcribed in a unique, cell-cycle-dependent pattern: *BLT1*, *BLT4*, and *BLT5* are strongly expressed but differentially regulated, *BLT2* and *3* are transcribed at low levels during sexual conjugation, and expression of *BLT6* occurs at low levels only during starvation ([25]; see also the *Tetrahymena* Gene Expression Database (TGED) at http://tged.ihb.ac.cn).

The microtubule cytoskeleton of *Tetrahymena* is amenable to genetic manipulation to analyze the incorporation and function of tubulins and/or MAPs *in vivo* [26]. In this report we employ molecular-genetic methods to compare the structural features and functions of two BLTs (BLT1 and BLT4) to those of the canonical BTU2. First, we show that BLT1 and BLT4 have evolved sequence changes in their GTP-binding sites, in their tubulin-tubulin interaction surfaces, and in their microtubule-targeting motifs that suggest that these noncanonical isotypes form distinct subsets of cellular, but non-axonemal, microtubules. Second, we employ transformation of the ciliate with expression vectors that direct the synthesis of Green Fluorescent Protein-tagged (GFP-tagged) versions of BLT1, BLT4, and BTU2 to dissect their incorporation into the microtubule cytoskeleton *in vivo*. We demonstrate that GFP-BLT1 and GFP-BLT4 participate in the formation of subsets of cortical microtubule structures in interphase cells that are distinct from those formed by GFP-BTU2. As predicted, GFP-BLT1 and GFP-BLT4 cannot be detected in somatic cilia and basal bodies, in striking contrast to GFP-BTU2. During cell division, GFP-BLT1 and GFP-BLT4, but not GFP-BTU2, are incorporated into the microtubule arrays of the macronucleus and into the mitotic spindle of the micronucleus. GFP-BLT1 also assembles into the microtubules of the micronuclear meiotic apparatus during conjugation. The differential partitioning of these isotypes is confirmed by biochemical fractionation and Western immunoblotting. We conclude that individual *Tetrahymena* β-tubulin isotypes can be used to construct subsets of microtubule structures that differ in cellular function, a result that strongly supports the multi-tubulin hypothesis. The diversity of microtubule structures formed by *T. thermophila,* a single-celled organism, provides an attractive model for dissecting the cellular mechanisms that underlie the selective sorting of tubulin isotypes.

## Results

### Amino acid sequences, structural motifs, and microtubule-targeting signals of *BLT1* and *BLT4*: Comparison to canonical *BTU2*

**Figure 1:**
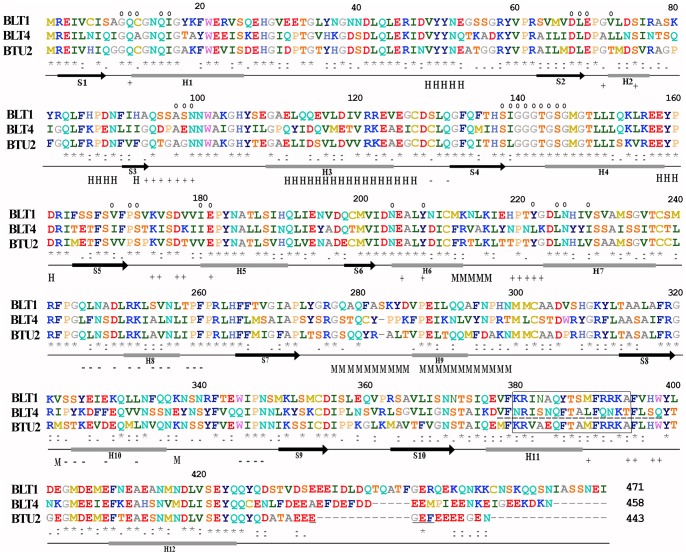
Sequence alignment of *T. thermophila* BLT1, BLT4, and BTU2 isotypes. The amino acid sequences of BLT1 (GenBank/TGD acc. no. TTHERM_01104960), BLT4 (acc. no. JQ979442), and BTU2 (acc. no. AAA30111.1) were aligned using ClustalW 1.83 [62]. Note that the BLT4 sequence possesses 21 amino acids (residues 378–398, underlined by dashes) encoded by the putative intron predicted *in silico* (GenBank/TGD acc. no. TTHERM_01120580), as deduced from the *BLT4* cDNA cloned in his study. Shown beneath the sequences are the secondary structural elements of the porcine β-tubulin monomer [28], [71]. Residues are colored according to their physicochemical properties following the RasMol Shapely scheme. In the sequence alignment, asterisks (*) indicate identical residues, colons (:) conserved substitutions, and periods (.) semi-conserved substitutions according to the Gonnet 250 matrix of ClustalW v1.83. Dashes (–) within the sequences indicate gaps introduced to maximize sequence similarity. Small circles (○) indicate residues involved in GTP-binding/hydrolysis, and H (helix H3) and M (M-loop) denote residues involved in lateral, interprotofilament contacts. Residues that participate in β/α (+ end) and α/β (– end) longitudinal interfaces are shown by + and −. The “axoneme motif,” EGEF, present in the C-terminal tail of BTU2 is underlined. Potential bipartite nuclear localization signals are boxed.

The sequences of the *BLT1* and *BLT4* genes are available from the *T. thermophila* Genome Annotation Database (TGD) under the accession numbers TTHERM_01104960 and TTHERM_01120580, respectively. To evaluate the conceptual predictions of these genes, we cloned and sequenced the *BLT1* and *BLT4* cDNAs. Although the genome annotation of the *BLT4* gene placed a putative intron at nucleotide positions 1129–1192 (relative to A = 1 of the initiator codon), the *BLT4* cDNA sequence indicated that the 63 nucleotides in question are not spliced out but rather encode 21 amino acids (Fig. 1, BLT4 residues 378–398 indicated by dashed underlining). We have referred this information to the TGD, which is revising the *BLT4* cDNA annotation.

#### Amino acid sequences

Figure 1 compares the predicted amino acid sequences of the 471-residue BLT1 and the 458-residue BLT4 isotypes to that of the canonical 443-residue BTU2 tubulin (GenBank acc. no. AAA30111.1). Excluding the divergent C-termini beyond residue 430, the amino acid sequences of BLT1 and BLT4 tubulins were 65% and 56% identical, respectively, to the sequence of BTU2, which corresponds to 140 and 180 residue substitutions in the noncanonical isotypes. BLT1 also contained a single amino acid insertion (Ser^283^). Table S1 shows the amino acid compositions of the three β-tubulins, of which three features are noteworthy: 1) the percentage of isoleucine in BLT4 was approximately twice that in BTU2 and BLT1 (10.1 *vs.* 4.6 and 5.9%, respectively); 2) the proportion of amino acids with small side chains was substantially decreased in the BLT4 isotype (44.4 *vs.* 51.3% for BTU2 and BLT1); and 3) BLT4 contained a higher percentage of amino acids with polar, uncharged side chains (30.8 *vs.* 25.8 and 25% for BTU2 and BLT1, respectively).

Cysteine and tyrosine residues in β-tubulins are, in general, highly conserved [2], yet four substitutions involving these amino acids distinguish the two *Tetrahymena* BLTs from BTU2. First, the conserved cysteine at position 12 was replaced by alanine in BLT4 but not in BLT1. Second, the cysteine at position 239 of BTU2 was substituted by threonine in the BLT4 isotype and serine in BLT1. Third, tyrosine at position 281 of BTU2 was replaced by cysteine in BLT4 and phenylalanine in BLT1. Finally, Tyr^425^ of BTU2 was replaced by cysteine in BLT4. Potential functional roles of these sequence alterations are considered in the Discussion.

Based on the compositional differences and the physicochemical characteristics of the contact surfaces of the noncanonical β-tubulins (see below and the Discussion), we hypothesize that BLT1 and BLT4 are not able to co-polymerize with BTU2 in individual microtubules. Next, we consider this hypothesis in the context of the structural motifs of β-tubulin.

#### Structural motifs: GTP-binding site and tubulin-tubulin interaction surfaces

**Figure 2:**
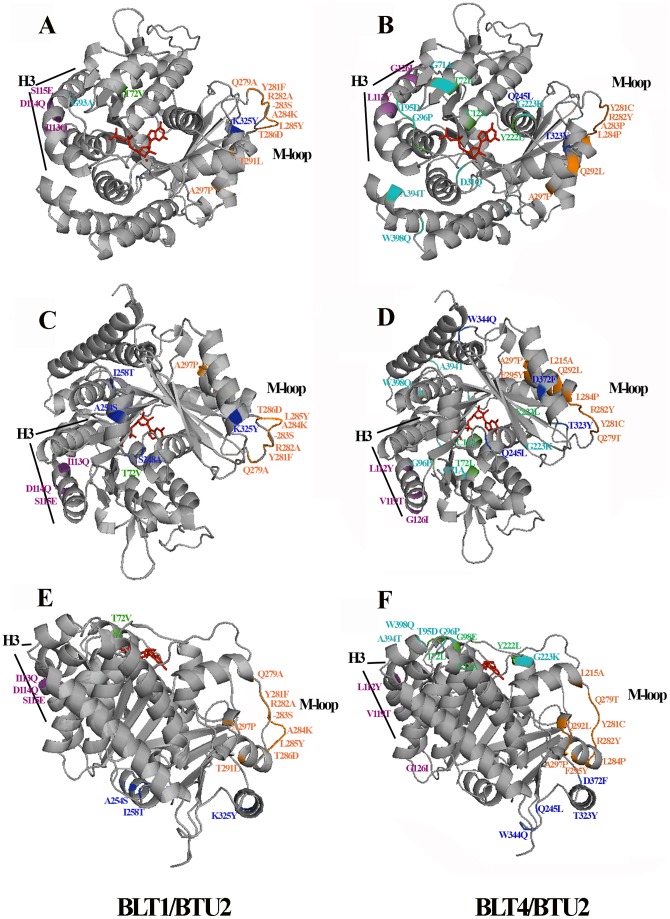
Residue substitutions in key structural motifs of the *T. thermophila* BLT1 and BLT4 isotypes. Ribbon diagrams of BLT1 (**A**, **C**, **E**) and BLT4 (**B**, **D**, **F**), superimposed on BTU2, are viewed from the plus end **(A**, **B)**, from the minus end **(C**, **D)**, and from outside the microtubule **(E**, **F)**. Nonconservative residue substitutions that distinguish BLT1 and BLT4 from BTU2: 1) in the GTP-binding/hydrolysis motif are shown in green; 2) at the plus- and minus-end surfaces [corresponding to the longitudinal interdimer (β/α) and intradimer (α/β) contacts] are colored in cyan and blue, respectively; and 3) at the lateral, interprotofilament contacts (principally H3 and the M loop) are denoted by purple and orange, respectively. The GTP molecule is shown in red.

Figure 2 shows three-dimensional models of *Tetrahymena* BLT1 (A, C, and E) and BLT4 (B, D, F) from three perspectives: 1) their plus ends (A, B); 2) their minus ends (C, D); and 3) their external surfaces (E, F). These views highlight the key structural motifs of the β monomer, the GTP-binding site and the tubulin-tubulin interaction surfaces, and show the residues substitutions that differentiate BLT1 and BLT4 from BTU2. Table 1 provides a concise summary of the alterations.

**Table 1:** Amino acid substitutions of BLT1 and BLT4 with respect to BTU2^a^.

Longitudinal interactions		Lateral interactions		GTP-binding site
Interdimeric (+) end	Intradimeric (−) end	H3	M-loop	
**G71A**	**Q245L**	**L112Y**	**T214V**	**C12A**
**G93(A)**	**S248(A)**	**I113(Q)**	**Q279(A)**	**T72(V)L**
**T95D**	**A254(S)**	**D114(Q)**	**Y281(F)C**	**G98E**
**G96P**	**I258(T)**	**S115(E)**	**R282(A)Y**	**Y222L**
**G98E**	**S323P**	**V119T**	**A284(K)P**	
**T178K**	**K325(Y)**	**G126I**	**L285(Y)P**	
**Y222L**	**D328F**		**T286(D)K**	
**G223K**	**W345Q**		**T291(L)K**	
**A394T**			**Q294L**	
**W398Q**			**F295Y**	
			**D296N**	
			**A297(P)P**	
			**K298(H)**	

a BTU2/residue/(BLT1)BLT4.

The GTP-binding sites of BLT1 and BLT4 were modified by substitutions relative to BTU2 (Fig. 2, Table 1). Although the γ-phosphate-binding motif (^140^GGGTGSG^146^) [27] was conserved among all three β chains, Figure 2 shows that the GTP pockets of BLT1 and BLT4 contained one and four residue substitutions [C12A, T72(V)L, G98E, Y222L; BTU2 residue/position/(BLT1)BLT4 residue, highlighted in green in Table 1], respectively, at positions that are otherwise highly conserved among the β-tubulins of most other organisms. These nonconservative substitutions change the charge and polarity of the GTP-binding pocket. The most striking of the four changes are the substitution of valine in BLT1 and leucine in BLT4 for threonine at residue position 72 and the replacement of Tyr^222^ by leucine in BLT4. The former is located near the entrance to the GTP-binding site, whereas the latter interacts by hydrogen bonding to the ribose 2′-OH and by π-π stacking with the guanine base (Fig. 2) [28], [29]. These changes may influence the binding affinity and hydrolysis of GTP and/or the egress of GDP and P_i_, factors that control the assembly and stability of microtubules [30] (for further details, see the Discussion).

The extensive sequence changes to the plus- (A, B) and minus-end (C, D) surfaces and in the H3 helices and M-loops (E, F) of the BLTs are readily apparent (Fig. 2, Table 1). For example, the M-loop of BLT1 is almost completely substituted with respect to BTU2, and that of BLT4 is substantially substituted. These replacements and the serine insertion at position 283 remodel the conformation of the BLT1 M-loop, but not the BLT4 loop, relative to the canonical β-tubulin. Because these four motifs undoubtedly retain their roles in longitudinal and lateral interactions but are highly substituted, α/BLT1 and α/BLT4 tubulin dimers may differ from α/BTU2 dimers in their capacities to form various classes of cellular microtubules.

#### Microtubule-targeting signals

Three features of BLT1 and BLT4 suggest that they serve a restricted set of non-axonemal functions in *Tetrahymena*. First, BLT1 and BLT4 lacked the C-terminal “axoneme motif” ^433^EGEF^436^ (underlined in Fig. 1) of canonical BTU2, which specifies incorporation of tubulin dimers into the singlet microtubules of the central pair of motile axonemes [14]. Second, the outer-arm dynein binding motif, ^55^(S/T)**G**(G/A)^57^ [16], which is present in BTU2, was not found in the noncanonical βLTs; the requisite glycine at position 56 was replaced by serine in BLT1, and the tripeptide found in BLT4, ^55^KAD^57^, bore no resemblance to the ^55^TGG^57^ of BTU2. Therefore, the BLTs are unlikely to recruit the binding of outer dynein arms. Third, the characteristic C-terminal motif for polyglutamylation and polyglycylation (^437^EEEE^440^), two posttranslational modifications that are essential to the function of the canonical BTU2 [31]–[33], was not discernible in the C-termini of BLT1 and BLT4. These sequence features suggest strongly that BLT1 and BLT4 do not participate in the formation of ciliary axonemes.

### Strategy for functional analysis of noncanonical and canonical β-tubulins

*In toto*, the divergent sequences and motifs of the noncanonical *Tetrahymena* BLT1 and BLT4 isotypes suggest that their regulatory regions for microtubule assembly and dynamics have evolved such that: 1) these isotypes, as partners in novel αβ dimers, may function independently of other β-tubulins to form distinct subsets of cellular, *but non-axonemal*, microtubules; and 2) they may require noncanonical α-tubulins to form tubulin dimers (not considered further herein). To address the first hypothesis, we generated somatically transformed *T. thermophila* cell lines that express GFP-tagged BLT1, GFP-tagged BLT4, or GFP-tagged BTU2 under the control of the cadmium-inducible promoter MTT1 (see Material and Methods). Following induction of fusion protein synthesis, synchronized cells were analyzed by epifluorescence microscopy. *During interphase, macronuclear amitosis, and micronuclear mitosis, GFP-BLT1 and GFP-BLT4 co-localized in identical microtubular structures,* ***which we illustrate here with GFP-BLT4*** ** ***and compare to the localization of GFP-BTU2*** *. In meiosis, however, BLT1 and BLT4 behaved differently.*

### Subcellular localization of *T. thermophila* BLTs and BTU2 during interphase

**Figure 3:**
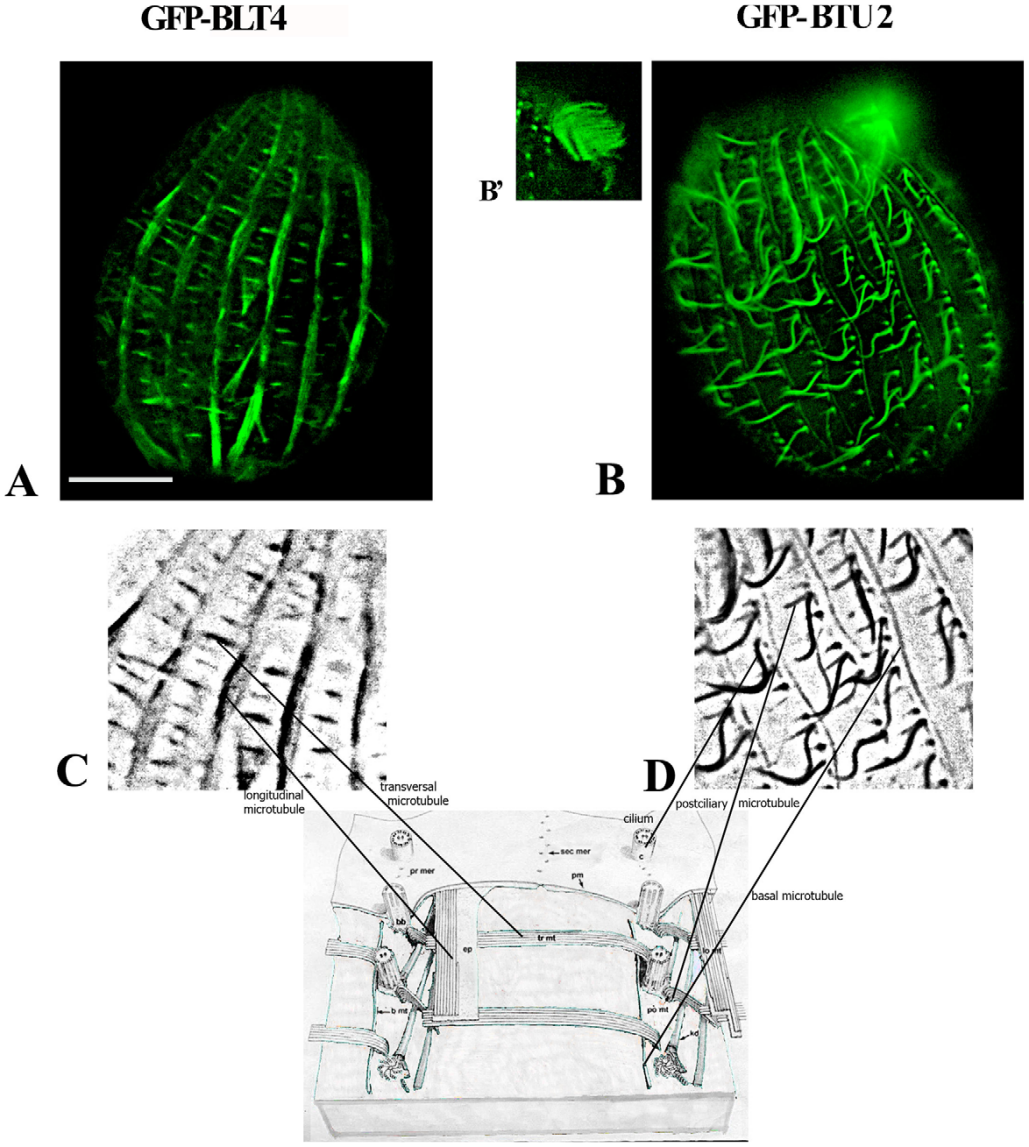
Cortical distribution of BTU2 and BLT4 in living *T. thermophila* cells during interphase. (**A, B**) Fluorescence microscopic images of the cortices of *T. thermophila* cells transformed with vectors encoding GFP-BLT4 or GFP-BTU2, respectively. (**B**') Labeling of the oral apparatus by GFP-BTU2. (**C**, **D**) Black-and-white enlargements of subregions from (A) and (B), respectively. Structural components of the microtubule cytoskeleton that incorporated GFP-BLT4 (A) or GFP-BTU2 (B) tubulins are keyed to the schematic representation of the subcortical cytoskeleton of a *Tetrahymena* cell (slightly modified from Allen [72]). GFP-BLT4 (and GFP-BLT1, not shown) was found in the longitudinal and transverse microtubule bundles (A, C), whereas GFP-BTU2 was present in somatic cilia, basal microtubules, and postciliary microtubules (B, D). Bar, 10 μm.

**Figure 4:**
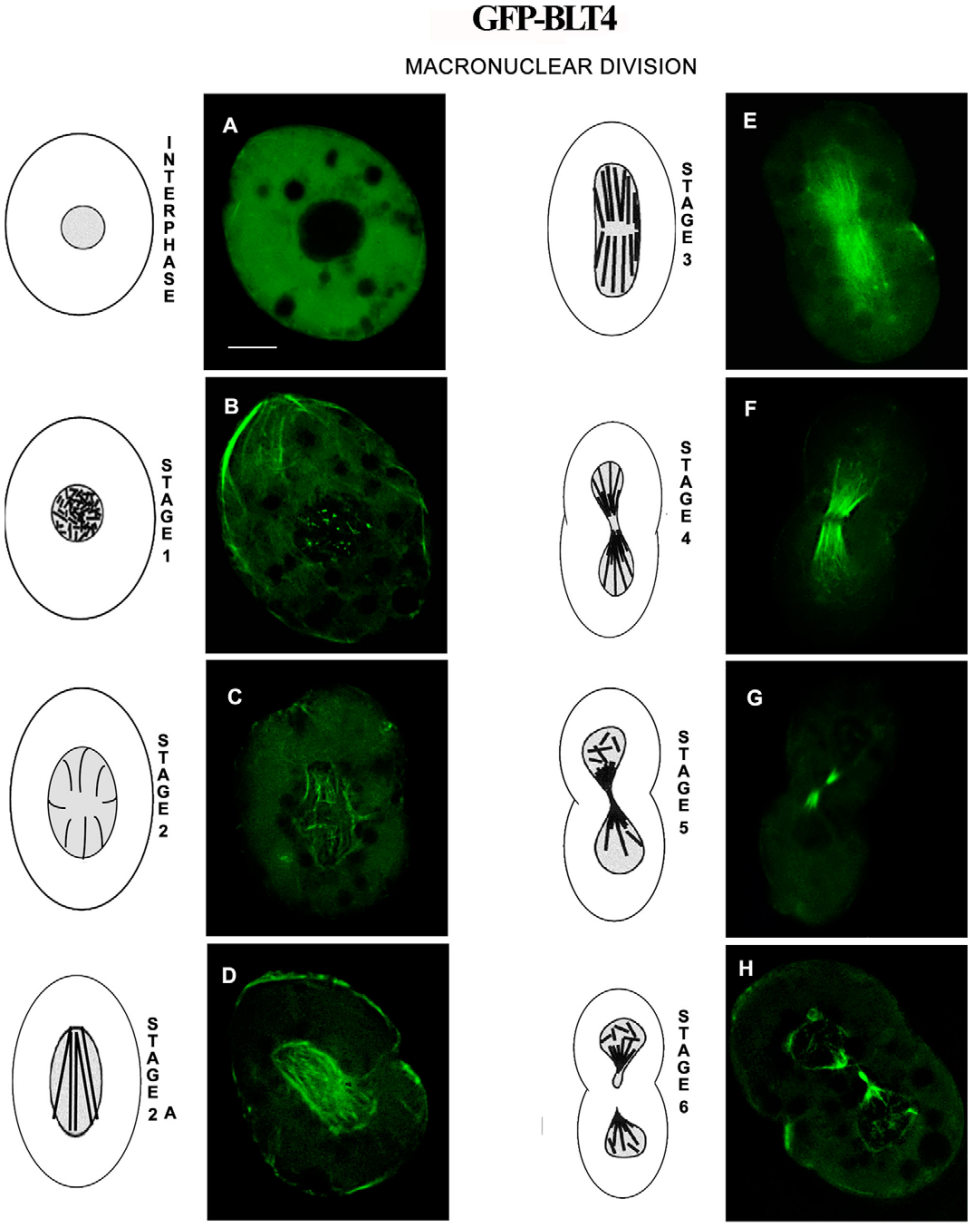
Distribution of BLT4 in *T. thermophila* cells during macronuclear division. (**A–H**) Fluorescence microscopic images of transformed *Tetrahymena* cells expressing GFP-BLT4 are shown for interphase (**A**) and for stages 1–6 of macronuclear division (**B–H**); interpretative drawings of each stage are positioned to the left of each micrograph. (A) In interphase, GFP-BLT4 was not detectable inside the nucleus. (B) At stage 1, GFP-BLT4 became incorporated into short, randomly oriented microtubules inside the macronucleus. (C) At stage 2, GFP-BLT4-labeled microtubules increased in length and projected from the center of the macronucleus toward its periphery. (D) At stage 2A, GFP-BLT4-labeled microtubules formed a fan-shaped structure inside the macronucleus. (E) At stage 3, the GFP-labeled macronuclear microtubules reorganized into a bifurcated, parallel array. (F) By stage 4, the cleavage furrow began to constrict the macronuclear membrane such that the central GFP-BLT4-labeled microtubules formed a “butterfly-like” pattern. (G, H) At stage 5, GFP-BLT4 was found on either side of the zone of macronuclear membrane constriction, and in stage 6, microtubules containing GFP-BLT4 were distributed to the two daughter macronuclei. The GFP-BLT1 isotype behaved identically to GFP-BLT4. Bar, 10 μm.

GFP-BLT4 labeled microtubules in two cortical structures, the longitudinal and transverse microtubule bundles (Fig. 3A, C), but were not present in somatic cilia. We did not detect GFP-BLT4 in the intracytoplasmic network, in the macronucleus (Fig. 4A), or in the micronucleus (data not shown) during interphase. In contrast, GFP-BTU2 was found, as expected, in cilia, in basal bodies, in the postciliary microtubules and basal microtubules of the cortex, and in the oral apparatus (Fig. 3B, B', D). Further consideration of these observations is reserved to the Discussion.

### Subcellular localization of *T. thermophila* BLTs and BTU2 during cell division

During cell division in ciliates, both the macronucleus and the micronucleus must divide. Macronuclear fission in ciliates occurs without the formation of a true mitotic spindle, a process called amitosis, but microtubules are nonetheless required [34], [35]. Micronuclear fission, by contrast, requires a conventional mitotic spindle. In both cases, fission takes place without the dissolution of the respective nuclear envelopes. Below we show that GFP-BLT4 participated prominently in these two nuclear divisions, whereas BTU2 did not.

#### BLTs and macronuclear fission

Fujiu and Numata [35] have described six morphological stages in *Tetrahymena* amitosis, which lasts ∼1 h, during which macronuclear microtubules undergo dynamic reorganization into distinct structures (see Fig. 4, stages illustrated adjacent to micrographs). During stage 1, GFP-BLT4 was incorporated into short, randomly oriented microtubules (Fig. 4B). At stage 2, the BLT4 isotype was present in longer microtubules that projected from the center of the macronucleus toward its periphery (Fig. 4C). This array then reorganized to form a fan-shaped structure (stage 2a; Fig. 4D) before resolving into the bifurcated parallel arrangement typically found in stage 3 (Fig. 4E). By stage 4, the cleavage furrow began constricting the macronuclear membrane such that the central GFP-BLT4-labeled microtubules formed a “butterfly-like” pattern (Fig. 4F). At stage 5, GFP-BLT4-labeled microtubules were found on either side of the zone of macronuclear membrane constriction (Fig. 4G). Upon completion of macronuclear fission (stage 6), microtubules containing GFP-BLT4 were distributed to the two daughter macronuclei (Fig. 4H). By the end of cytokinesis, the macronuclear microtubules had disassembled, restoring the condition shown in Figure 4A.

#### BLTs and micronuclear fission

**Figure 5:**
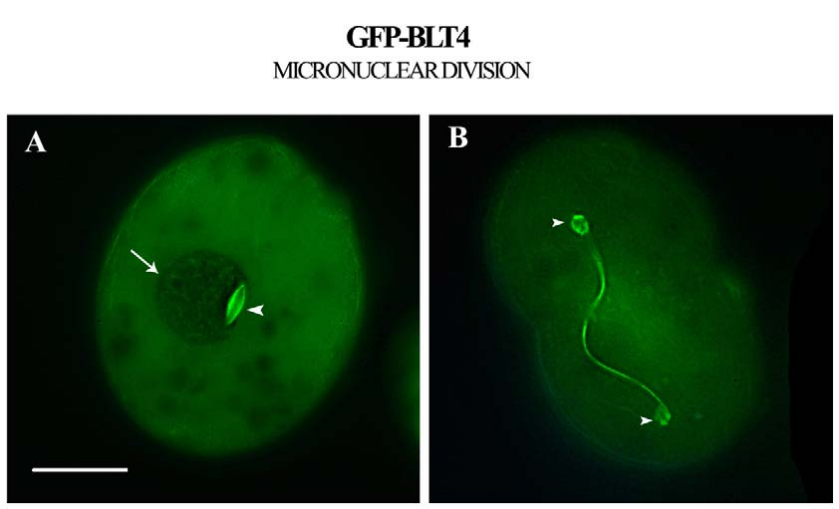
Distribution of BLT4 in *T. thermophila* cells during micronuclear division. (**A**) GFP-BLT4 labeled microtubules of the metaphase mitotic spindle of the micronucleus (arrowhead) which lies adjacent to the macronucleus (arrow). (**B**) During anaphase and telophase, the mitotic spindle, composed of 13-protofilament, 24-nm microtubules, depolymerized and the tubulin dimers were recycled to generate the 14–16 protofilament, 27–32-nm microtubules of the separation spindle, which elongated 10-fold to distribute the newly formed micronuclei (arrowheads) to the two daughter cells. Bar, 10 μm.

Figure 5A shows that GFP-BLT4 was present in microtubules of the metaphase mitotic spindle of the micronucleus, which resides adjacent to the macronucleus. During anaphase and telophase, the mitotic spindle reorganized to form the separation spindle, which increases 10-fold in pole-to-pole length to distribute the newly formed micronuclei to the two daughter cells (Fig. 5B). The microtubules of the separation spindle retained GFP-BLT4 throughout the transition, consistent with recycling of this isotype from the depolymerizing, 13-protofilament microtubules of the metaphase spindle to the elongating, 14–16 protofilament microtubules of the separation spindle (see Discussion), as originally proposed for *Paramecium* [36].

#### GFP-BTU2 tubulin during somatic cell division

**Figure 6:**
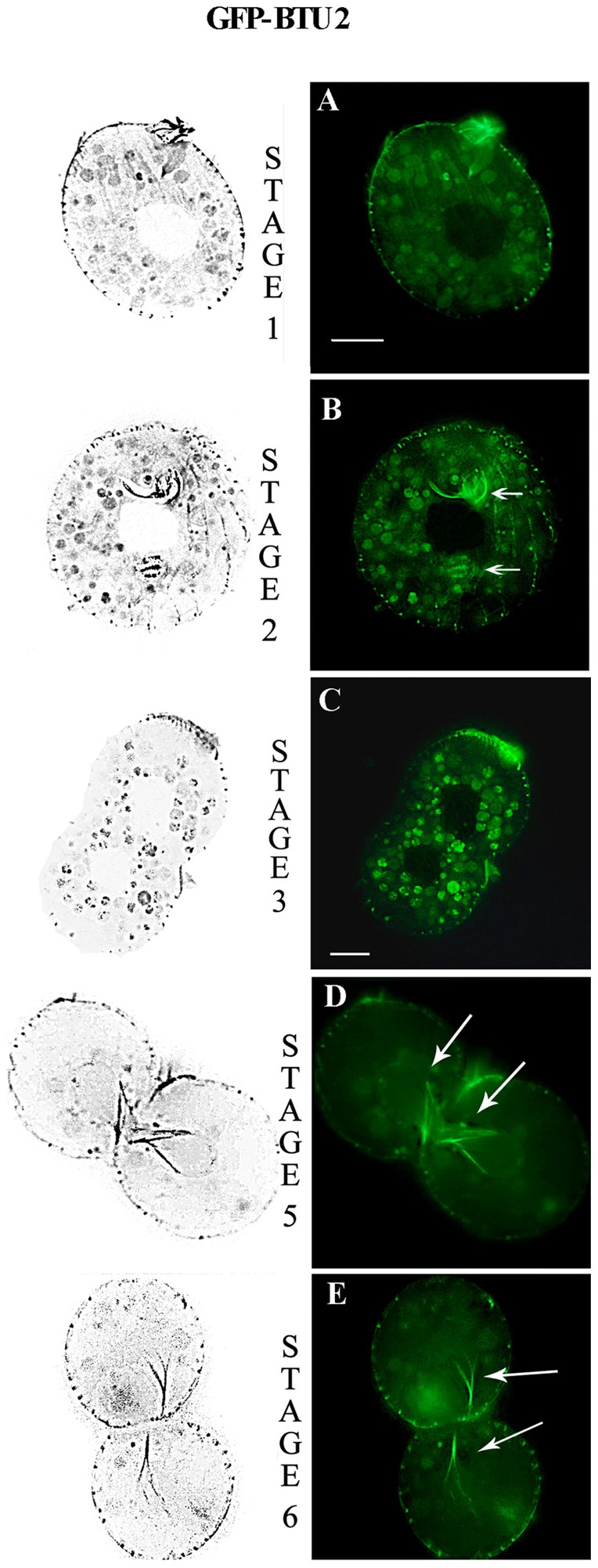
Distribution of BTU2 in *T. thermophila* cells during macronuclear division. (**A–E**) Fluorescence microscopic images of transformed *Tetrahymena* cells expressing GFP-BTU2. GFP-BTU2 was found in basal bodies at the cell cortex during stages 1–6 (stages 1, 2, 3, 5, and 6 shown here) and in the newly formed oral apparatus (A, B; see arrows). Autofluorescent food vacuoles can be observed in (A–C). As cytokinesis proceeded, the canonical β-tubulin was observed in two sets of microtubules that crisscrossed the constriction furrow between the dividing macronucleus (D, E; see arrows). Micrographs were recorded from different cells at the cell cycle stages specified. Bar, 10 μm.

We did not detect GFP-BTU2 tubulin either in the microtubule arrays of the macronucleus or in the mitotic and separation spindles of the micronucleus during cell division. Rather, GFP-BTU2 was found in basal bodies at the cell cortex during stages 1–6 (Fig. 6A–D) and in the newly formed oral apparatus (Fig. 6A, B; stages 1 and 2, see arrows). As cytokinesis proceeded (stages 5 and 6), the canonical β-tubulin was observed in two sets of microtubules that crisscrossed the constriction furrow between the dividing macronucleus (Figs. 6D, E; see arrows), consistent with the possibility that they generate force to facilitate nuclear separation by acting outside of the persistent macronuclear envelope. Finally, GFP-BTU2 was present in cilia, in postciliary microtubules, and in basal microtubules as described previously for interphase (Fig. 3); these structures were not captured in Figure 6 due to the location of the microscopic focal plane deep within the *Tetrahymena* cells.

### Subcellular localization of *T. thermophila* BLTs and BTU2 during conjugation

Conjugation is the sexual stage of the *Tetrahymena* life cycle, during which two cells pair, form a temporary junction, exchange gamete nuclei, and generate the new micronuclei and macronuclei of their progeny. The nuclear events of conjugation include meiosis within the micronuclei, gamete nuclei formation, fertilization by gamete nuclear exchange between the two paired cells, and two postzygotic micronuclear mitoses by each daughter cell. The spindles, whether meiotic or mitotic, always reside within the micronuclei.

**Figure 7:**
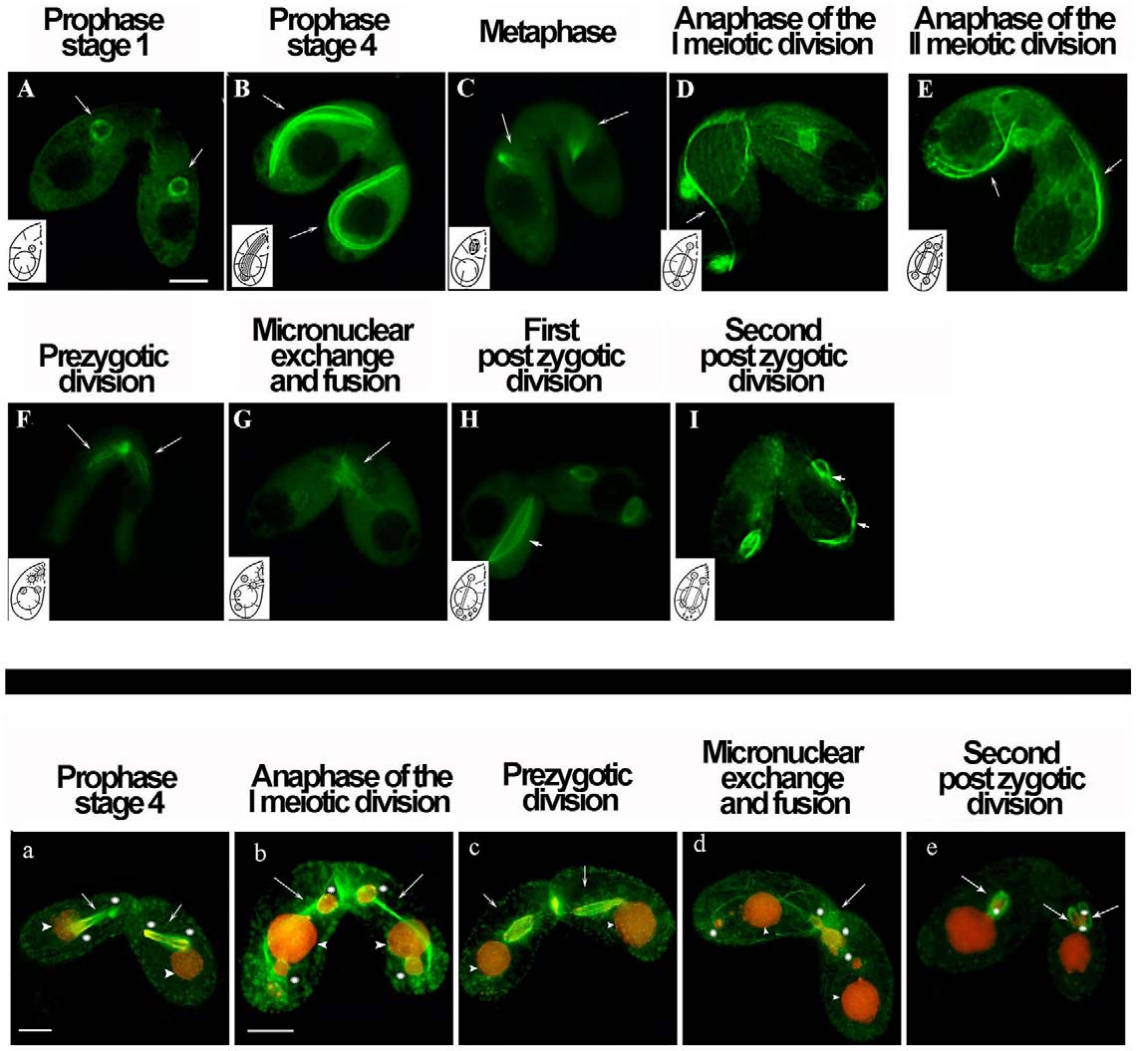
Distribution of BLT1 during conjugation. Upper panel: Fluorescence microscopic images of *Tetrahymena* cells expressing GFP-BTL1 *in vivo*. To assist interpretation, each image contains an inset that schematically represents the microtubule structures present during the main stages of conjugation as described by Gaertig and Fleury [73]. **Bottom panel**: Fluorescence microscopic images of *Tetrahymena* cells expressing GFP-BTL1 after fixation and nuclear staining with propidium iodide; the micronuclei (asterisks) and macronuclei (arrowheads) of the conjugants stained red. (**A**) *Tetrahymena* cells shortly after formation of the conjugating pair (stage 1 of prophase). GFP-BTL1 was visible in the micronuclei (arrows), whereas the macronuclei were negative for GFP-BLT1 fluorescence. (**B** and **a**) During prophase stage 4 of the first meiotic division, GFP-BLT1 forms curved microtubule bundles within the elongating micronucleus (arrows). (**C**) At metaphase of meiosis I, GFP-BLT1 was observed in normal spindles (arrows). At anaphase of the first (**D** and **b**) and second (**E**) meiotic divisions, metaphase spindles depolymerized and their tubulin dimers were used to form elongating separation spindles (arrows). (**F** and **c)** Four haploid nuclei were formed, one of which underwent a mitotic division (prezygotic mitosis) as shown by the GFP-BLT1 fluorescent signal and indicated by the arrows; the three remaining haploid meiotic products degenerated (not shown). (**G** and **d**) Subsequently, GFP-BLT1 is found in the micronuclei that exchanged between mating partners, and in the mitotic spindles of the first and second postzygotic divisions (**H,** **I** and **e**). (**H** and **I)** The conjugating cells are slightly asynchronous in the first and second postzygotic divisions such that elongated mitotic spindles are visible in only one member of each pair. Micrographs were recorded from different cells at the conjugation stages specified. Bars, 10 μm.

Figure 7 shows the localization of GFP-BLT1 in the micronuclei of conjugating cells *in vivo* (A–I) and after propidium iodide staining of fixed cells to better visualize nuclear behavior (a–e). Shortly after the formation of the conjugating pair, α/GFP-BLT1 dimers formed curved, intranuclear microtubule bundles (Fig. 7A) that elongated dramatically (Fig. 7B) as the micronuclei expanded around the macronuclei (prophase stages 1, 4). These bundles (green) overlapped with the micronuclear chromatin (red) as shown by the yellow zones (Fig. 7a), consistent with the intranuclear location of meiotic prophase microtubules described by Wolfe et al. [37]. At metaphase of meiosis I, conventional, GFP-BLT1-labeled spindles were observed (Fig. 7C), and these spindles elongated to form separation spindles during anaphase of meiosis I (Figs. 7D and 7b). As meiosis proceeded through the second division (Fig. 7E), four haploid nuclei were produced; three degenerated (not shown), whereas the fourth haploid nucleus of each conjugating cell divided mitotically to yield two identical haploid pronuclei (Figs. 7F and 7c). Subsequently, the mating partners reciprocally exchanged one of their two pronuclei. GFP-BLT1 was present both in the exchanging micronuclei (Fig. 7G and 7d) and in the mitotic spindles of the first and second postzygotic divisions (Figs. 7H, 7I and 7e). One of the two newly formed micronuclei will generate a new macronucleus, whereas the parental macronucleus will degenerate (not shown). Throughout meiosis, GFP-BLT1 was also detected in the longitudinal and transverse microtubule bundles (Fig. 7D and 7a). However, GFP-BLT1 was not detected in the intracytoplasmic network and in somatic cilia, as during interphase.

**Figure 8:**
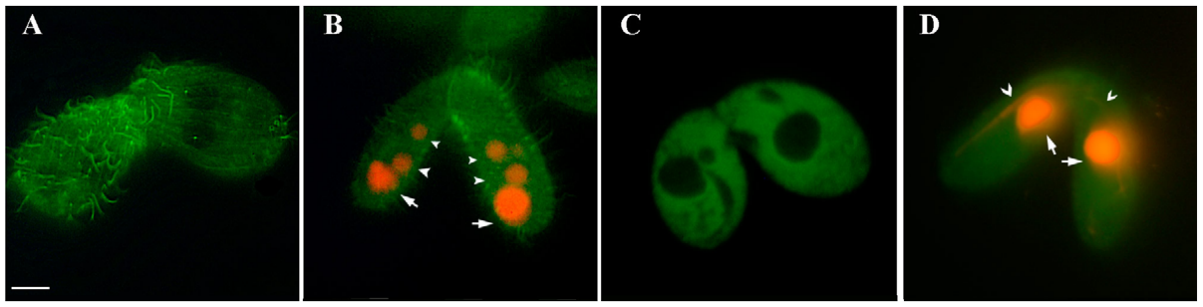
Distribution of BLT4 and BTU2 during conjugation. Fluorescence microscopic images of *Tetrahymena* conjugant cell pairs expressing GFP-BTU2 (**A** and **B**) or GFP-BTL4 (**C** and **D**). (A) and (C) are micrographs of living cells, whereas (B) and (D) are images of cells after fixation and staining with propidium iodide. In (A) and (C), the nuclei are negative for GFP-tubulin fluorescence. (B) Propidium iodide stained the newly formed micronuclei (arrowheads) and the parental macronuclei (arrows) in conjugants at the first post-zygotic division. (D) Propidium iodide stained the macronuclei (arrows) and the elongating micronuclei (arrowheads) in conjugants during anaphase of meiosis I. Although GFP-BTU2 and GFP-BLT4 did not label nuclear microtubules during meiosis, GFP-BTU2 was visible in cilia of the conjugant cells (A and B). Micrographs were recorded from different cells at the conjugation stages specified. Bar, 10 μm.

In contrast to BTL1, BTU2 and the BLT4 were not detected in the microtubules that participated in meiosis and nuclear exchange (Fig. 8). The micronuclei and macronuclei of GFP-BTU2- and GFP-BLT4-transfected cells (Fig. 8A, B and C, D, respectively) showed no evidence of incorporation of these GFP-tagged tubulins. Nevertheless, GFP-BTU2 (Fig. 8A, B) was present in cilia and basal bodies, consistent with our prior observations of interphase (Fig. 3) and mitotic (Fig. 6) cells.

### Biochemical analysis of the subcellular localization of *T. thermophila* BLT1, BLT4, and BTU2

**Figure 9:**
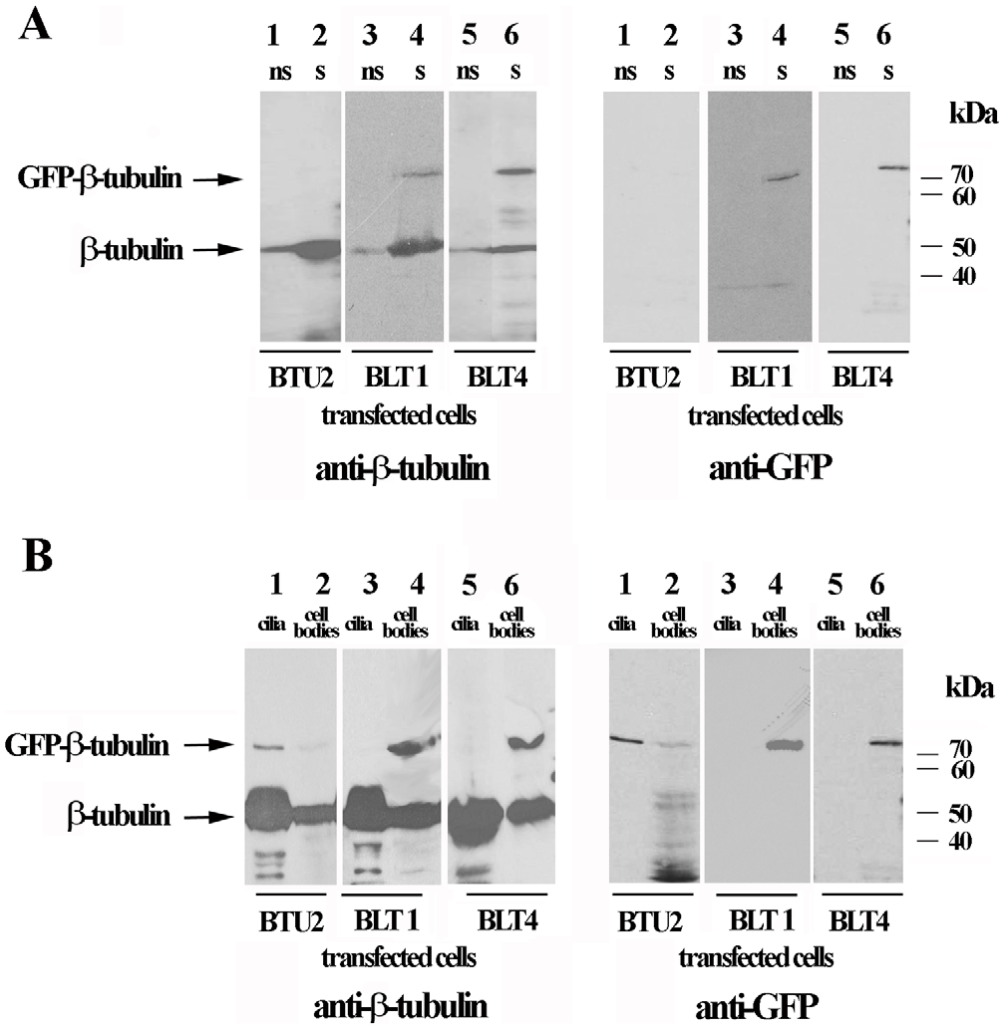
Analysis of the subcellular localization of the BLT1, BLT4, and BTU2 isotypes by biochemical fractionation and Western blotting. (**A**) Nuclei were purified from non-synchronous cells (ns, lanes 1, 3, and 5) and from synchronously dividing cells (s, lanes 2, 4, and 6) after induction of GFP-BTU2, GFP-BLT1, or GFP-BLT4 synthesis (see Material and Methods). After samples were subjected to SDS-PAGE and electrotransfer to nitrocellulose, blots were developed with anti-β-tubulin or anti-GFP primary antibodies and appropriate secondary antibodies (Materials and Methods). The “anti-β-tubulin” panel contains lanes derived from two different blots, whereas the “anti-GFP” panel derived from the same blot. (**B**) Cilia were separated from *Tetrahymena* cell bodies after induction of GFP-BTU2 or GFP-BLT4. Blots were developed as for (A). **Results**: GFP-BLT1 and GFP-BLT4 were predominantly found in samples containing nuclei purified from synchronously dividing cells (panel A, lanes 4 and 6) or in deciliated cell bodies (panel B, lanes 4 and 6). In contrast, GFP-BTU2 was present mainly in purified cilia (panel B, lanes 1).

Based on the results presented thus far, we conclude that BLT1 and BLT4 are used primarily to form microtubules that function in macronuclear amitosis and micronuclear mitosis, that BLT1 is also involved in micronuclear meiosis, and that BTU2 is largely restricted to forming ciliary microtubules and basal bodies. From these conclusions we derive two predictions: 1) GFP-BLTs should be enriched in nuclei from cells in synchronous culture relative to nuclei from asynchronous cell cultures, whereas GFP-BTU2 should be absent from nuclear preparations; and 2) recovery of GFP-BTU2 should be enhanced in cilia relative to cell bodies following deciliation of *Tetrahymena* cells expressing the canonical isotype, whereas the converse should be true for cells expressing GFP-BLT1 and GFP-BLT4. To test these predictions, we transformed three identical cultures of *Tetrahymena* with the GFP-BLT1, GFP-BLT4 and GFP-BTU2 vectors and then characterized the distribution of the three β-isotypes in cells by biochemical fractionation and Western immunoblotting. After cadmium-induced synthesis of the GFP-tagged β chains, *Tetrahymena* cells were processed to generate nuclei from non-synchronous *Tetrahymena* cultures or from synchronous cells in division (Fig. 9A) or to obtain purified cilia and deciliated cell bodies (Fig. 9B). After SDS-PAGE of the samples and transfer of proteins to nitrocellulose membranes, duplicate blots of the two experimental preparations were probed either with a monoclonal antibody against β-tubulin or with a polyclonal antibody specific for GFP. As shown in Figure 9, we obtained strong immunoreactive bands corresponding to GFP-BLT1 and GFP-BLT4 in the samples containing nuclei purified from synchronously dividing cells (Fig. 9A, lanes 4 and 6) or composed of deciliated cell bodies (Fig. 9B, lanes 4 and 6). Furthermore, the GFP-BLTs were not detected in nuclear extracts from unsynchronized cells (Fig. 9A, lanes 3 and 5) or in samples of purified cilia (Fig. 9B, lanes 3 and 5). In contrast, GFP-BTU2 was present in ciliary fractions (Fig. 9B, lanes 1), and a faint signal was detectable in the cell body fraction (Fig. 9B, lanes 2). However, GFP-BTU2 was absent in nuclear fractions, whether obtained from synchronized or non-synchronized cells (Fig. 9A, lanes 1 and 2). These results confirmed that the noncanonical BLTs participate in forming microtubules involved in mitosis, whereas a major function of the canonical BTU2 is to participate in assembly of ciliary microtubules.

## Discussion

Prior to sequencing of the *T. thermophila* genome, it was thought that single α- and β-tubulin isotypes sufficed, with various posttranslational modifications, to form the many different microtubule systems of this unicellular organism [18], [19], [21], [22]. However, completion of the genome led to the discovery of novel *α-* and *β-tubulin* genes, including three and six genes encoding noncanonical α-like and β-like tubulins, respectively [24]. These noncanonical *tubulin* genes raised the possibility that the encoded tubulin isotypes may be used to form functionally distinct subsets of microtubules. Here we report the cloning of two novel *β-like tubulin* (*BLT1* and *BLT4*) cDNAs and show that they encode divergent 471- and 458-residue β-tubulin isotypes whose interaction surfaces and GTP-binding sites are substantially altered relative to canonical BTU2 (a single isotype encoded by two synonymous genes) by numerous nonconservative amino acid substitutions. We also examine the function of these noncanonical β-tubulin isotypes with respect to that of BTU2 by transforming *T. thermophila* cells with expression vectors that direct the inducible synthesis of GFP-tagged variants of the three isotypes. Our results demonstrate that the BLTs function *within nuclei* during macronuclear amitosis and micronuclear mitosis and in two cortical microtubule structures, the longitudinal and transverse microtubule bundles. Furthermore, BLT1, but not BLT4, is involved in meiosis during the sexual conjugation. Canonical BTU2, by contrast, is found in cilia, basal bodies, two other cortical structures (the postciliary microtubules and basal microtubules), and in the bundle of microtubules that forms *outside* the macronucleus during macronuclear division. Therefore, we conclude that the BLT and BTU2 isotypes are used in distinct microtubule subsets, a result that provides strong support for the multi-tubulin hypothesis.

One may legitimately question whether the GFP-tagged BLTs that we have used in our studies accurately report the functionality of the corresponding wild-type *Tetrahymena* proteins. The expression of α- and β-tubulins containing GFP fused in frame to their N- or C-termini has been successfully used to track microtubule dynamics and reorganization in mammalian cell lines, fish and insect embryos and cell lines, fungi, plants, and protists, including *Tetrahymena* [38]–[44]. The few cases in which GFP-tagged tubulins alter microtubule phenotypes appear to be system-specific [38]. We observed no perturbations of microtubule behavior by incorporation of GFP-tagged BLT1, BLT4, or BTU2 into their respective microtubule subsets, and the growth, somatic division, and sexual conjugation of *Tetrahymena* cells expressing the tagged tubulins did not differ from the wild-type. We conclude that incorporation of tagged *Tetrahymena* β-tubulins into cellular microtubules provides a valid read-out of β-isotype functionality.

### Distinctive features of the primary sequences of the BLTs and BTU2 isotypes

The amino acid sequences of BLT1 and BLT4 differ from BTU2 at 140 and 180 residue positions (excluding the C-terminus), respectively, and many are nonconservative substitutions that are located in otherwise highly conserved regions: 1) that contribute to tubulin-guanine nucleotide interactions; 2) that participate in tubulin-tubulin contacts; or 3) that target β-tubulins to distinct microtubule structures. Although these regions (and others) undoubtedly interact synergistically to establish the functional properties of tubulin and microtubules, consideration of them separately provides a convenient framework for interpreting and integrating our results with the literature on tubulin structure/function relationships. Table 1 summarizes the substitutions discussed below.

Several important amino acid substitutions (Table 1; green) occur in sequence motifs of the BLT1 and BLT4 isotypes that contribute to the GTP-binding site and that are otherwise conserved in most β-tubulins, including BTU2. These changes (C12A, T72(V)L, G98E, Y222L; BTU2 residue/position/(BLT1)BLT4 residue) alter the charge and polarity of the GTP-binding/hydrolysis site. Replacements at two of the positions, valine (BLT1) or leucine (BLT4) for tyrosine at position 72 and leucine for tyrosine at position 222 (BLT4), may be particularly important (Fig. 2). The first positions a large, hydrophobic side chain near the entrance to the BLT4 GTP-binding pocket, whereas the second eliminates important molecular interactions that orient the nucleotide in its binding site [28], [29]. We propose that the residues at BLT positions 72 and 222 may control the energetics and kinetics of the β-tubulin GTP cycle, thereby conferring differential polymerization properties on distinct α/BLT dimers. Site-directed mutagenesis of these two positions – singly, paired, and in combination with the two other substituted sites of the *T. thermophila* BLT4 GTP pocket – should enhance our understanding of the molecular regulation of GTP binding and hydrolysis by β-tubulins.

Both the longitudinal and lateral contact surfaces of BLT1 and BLT4 show extensive remodeling with respect to those of BTU2 (Fig. 2). The interdimer surfaces of the BLTs tend to be more polar and charged, whereas their intradimer contacts are slightly more hydrophobic (Table 1). Substitutions found in the lateral interaction surfaces of the BLTs, which are formed by helix H3 and the M-loop, do not show obvious patterns of bias in the physicochemical properties of their amino acid side chains. Nonetheless, it is clear that the sequences of H3 and the M-loop have diverged substantially from those of the canonical β-tubulins of many protozoans. We suggest that the interdimer and lateral contact surfaces of the two BLTs are sufficiently divergent from those of BTU2 that the former cannot co-assemble into microtubules with the latter. Nonetheless, BLT1 and BLT4, which are also markedly divergent in sequence, are capable of co-assembly in subsets of microtubules in interphase, micronuclear mitosis, and macronuclear amitosis. We cannot rule out the possibility that incorporation of the BLT and BTU2 isotypes into distinct sets of microtubules results from isotype-specific transport mechanisms as yet unknown.

The locations of cysteine and tyrosine residues in β-tubulins are generally conserved [2]. The replacement of Cys^239^ by Thr in BLT4 is a rare example of this hydroxyl amino acid at this position; substitution of serine, as in BLT1, has been observed in fungal and some protistan β-tubulins [2], [45], [46] and in vertebrate β-tubulin isotypes III and V [1]. Furthermore, BLT4 has alanine replacing cysteine at position 12. With respect to BTU2, the C239T and C12A substitutions in BLT4 are caused by two and three base changes, respectively, to the corresponding codons [codon 239, TGT (Cys) to ACT (Thr); codon 12, TGT (Cys) to GCA (Ala)]. Therefore, these rare substitutions at highly conserved positions must be under strong positive selection pressure, which may require compensatory amino acid changes elsewhere in the protein [29]. Joe et al. [1] have proposed that Ser^239^ is particularly important to maintain a conformation of the vertebrate βIII tubulin isotype that is compatible with: 1) efficient incorporation into microtubules; 2) appropriate control of microtubule dynamics; and 3) interaction of microtubules with other proteins. The substitutions of cysteines for conserved tyrosines at positions 281 and 425 of BLT4 are also highly unusual, but are the result of single base changes. Y281C and Y425C, which are located in the M-loop and near the C-terminus, respectively, were found previously in one of four β-tubulin isotypes from an Antarctic ciliate, *Euplotes focardii* [45], [46]. Therefore, understanding the functional significance of the unusual interchange of aliphatic/phenolic hydroxyl- and thiol-containing amino acids at positions of high conservation in β-tubulins may reveal unappreciated subtleties of tubulin-tubulin interactions and microtubule dynamics.

### Functional roles of BLTs and BTU2 in *Tetrahymena*

*Tetrahymena* BLT1 and BLT4 lack the structural motifs associated with tubulin incorporation into the axonemal central pair, the binding of outer-arm dynein, and the addition of C-terminal polyglutamyl and/or polyglycyl posttranslational modifications [14], [16], [31], [32], [47], whereas these motifs are present in BTU2 (Fig. 1). Thus, we hypothesized that the two noncanonical βLTs are used to form *non-axonemal* microtubules that mainly function in dynamic cellular structures rather than in stable microtubule systems. The results of our genetic transformation and biochemical fractionation experiments provide substantial support for this hypothesis.

When *Tetrahymena* cells are transformed with expression constructs that support the inducible production of GFP-tagged β-tubulins, the BLT and BTU2 isotypes segregate into different subsets of microtubules. During cell division, BLTs are incorporated into dynamic microtubules specifically required for nuclear division, whether mitotic and meiotic (i.e., micronuclear) or amitotic (macronuclear). Both BLTs participate in somatic micronuclear division, but only BLT1 is used for meiotic divisions. BTU2, by contrast, is primarily found in microtubules of relatively stable structures (e.g., the ciliary axoneme and basal bodies) in interphase, mitosis, amitosis, or meiosis; one exception is its inclusion in the microtubules that appear to constrict the furrow of the dividing somatic micronucleus. All three β-isotypes are used to form cortical microtubule structures, BLT1 and BLT4 in the longitudinal and transverse microtubule bundles and BTU2 in postciliary and basal microtubules. Thus, we have demonstrated that three of the seven β-tubulin isotypes, two noncanonical and the other canonical, are differentially incorporated into ten of the 17 microtubule structures described in this organism (reviewed by Gaertig [18]).

Our biochemical fractionation experiments support the conclusions drawn from our microscopic observations. GFP-BLTs are enriched in nuclear preparations from synchronously dividing cells relative to asynchronous cell cultures and are found in deciliated cell bodies but not in cilia themselves. GFP-BTU2 is detected in ciliary fractions, is present at low levels in cell bodies, but is absent from all nuclear fractions. Thus, the three isotypes partition biochemically as predicted by our *in vivo* experiments.

Smith et al. [48] have described a mutation, *btu1-1*, that uncouples macronuclear and micronuclear division from cytokinesis in *Tetrahymena*. The BTU1 isotype expressed by the *btu1-1* allele contains a methionine substitution for lysine at position 350 and functions in a dominant-negative fashion over the wild-type protein produced by the *BTU2* locus. The macronuclear phenotype of the mutant entails formation of microtubule bundles that surround the macronucleus but do not migrate to the cleavage furrow. Micronuclei, by contrast, form long anaphase spindles that persist well beyond the initiation of cytokinesis. These phenotypes cannot be explained by an intranuclear effect of the mutant protein, because we have shown that BTU2, which is identical to BTU1, does not enter either the macronucleus or the micronucleus. Thus, we conclude that the mutant protein, BTU1-K350M, perturbs nuclear division by affecting furrow-associated microtubules *outside* of the nucleus. Intranuclear microtubules of the macronucleus and the micronucleus incorporate BLT1 and BLT4 but not BTU2. Whether other BLTs also function within the nucleus remains to be investigated.

The distinct subcellular localizations and functions of *Tetrahymena* BLTs appear to be controlled by intrinsic structural features of their primary structures and/or conformations rather than by posttranslational modifications. For example, we show here that the BLTs participate in forming both the metaphase spindle and the separation spindle as the *Tetrahymena* micronucleus divides. In *Tetrahymena* [49] and in other ciliated species (e.g., *Paramecium* and *Nyctotherus* [36], [50]) the metaphase spindle, which is composed of 13-protofilament, 24-nm microtubules, largely depolymerizes as the separation spindle, which contains 14–16 protofilament, 27–32-nm microtubules, undergoes elongation through tubulin incorporation. We propose that BLT1 and BLT4 increase the elasticity of the lateral interactions between protofilaments, based on substitutions in their M-loops and H3 helices, such that protofilament numbers in excess of the conventional 13 can be accommodated as tubulin dimers are recycled from the metaphase spindle to the separation spindle. It is unlikely that posttranslational polyglutamylation and/or polyglycylation of the BLTs could facilitate a direct conversion in protofilament number during micronuclear spindle reorganization because neither BLT1 nor BLT4 possess the C-terminal motif for these modifications (EEEE) and because these modifications are added to tubulin subunits only *after* their incorporation into microtubules [51]. However, we note that posttranslational modifications of the BLTs have yet to be studied in *Tetrahymena*.

BLTs function within both the macronucleus and the micronucleus during somatic cell division. Because GFP-BLTs are not detectable within the nuclei during interphase, they must be transported across the nuclear membrane at the start of cell division, which suggests that this isotype might possess a nuclear localization signal (NLS) sequence. BTU2 and BLT1, *but not* BLT4, contain a potential bipartite NLS, ^380^ **KR**(V/I)(A/N)(E/A)Q(F/Y)T(A/S)MF**RRK**A^394^, with two basic amino acids (bold font) separated by a nine-residue spacer from three additional basic residues (bold); this sequence is substantially altered in BLT4 (Fig. 1, boxed). Many β-tubulins contain similar putative NLSs, but there is no evidence that they function in translocation of tubulin to the nucleus. One may reasonably ask, “why not?” Dingwall and Laskey [52] have shown that three features of a bipartite NLS are critical for function. First, no more than two basic amino acids can be accommodated in the first segment, whereas the second can have three to five. Second, the two clusters of basic residues must be separated by a minimum of 10 amino acids. Third, the NLS basic peptides must be presented to its receptor in extended, β-strand conformation. Therefore, the explanation for the failure of the conserved, NLS-like motifs of β-tubulins to dictate nuclear localization is clear – the second and third criteria are not met. The nine amino acid spacer of the β-tubulin sequence is too short and is located within helix 11 on the surface of the folded chain. As an alternative hypothesis for nuclear localization of a β-tubulin isotype, mammalian βII, Walss-Bass et al. [53] have proposed that αβII dimers “hitchhike” as specific passengers on other proteins that do contain an NLS or that αβII dimers specifically co-assemble with the nucleus at the end of mitosis. One plausible mechanism for nucleus-specific import, or regulated hitchhiking, of proteins has emerged with the recognition that the micronucleus and macronucleus of *Tetrahymena* possess distinct nucleoporins [54], [55], the “gatekeepers” of nuclear pores, and importin α “adaptors” [55], which bind to specific cargo proteins (reviewed by Orias et al. [56]). Our results favor a nucleoporin/importin-mediated mechanism for nuclear localization of *Tetrahymena* BLT1 and BLT4 tubulin. The use of BLT1, but not BLT4, in micronuclear meiosis may be due to differential expression of their genes, which causes a substantial increase in BLT1 mRNA synthesis at this stage of conjugation [25]. With its suite of molecular-genetic tools, the model protozoan *T. thermophila* provides an ideal test bed for evaluation of potential mechanisms of nuclear import of β-tubulins used in mitosis and/or meiosis.

We did not detect the BLTs or BTU2 in the intracytoplasmic microtubule network. This may be a valid observation, but our experimental protocol may have prevented detection due to the moderate fluorescent background generated by unpolymerized pools of the GFP-tagged isotypes, to low levels of incorporation of these β isotypes into the complex, or to both.

Tubulin subunits must interact with a wide variety of other proteins in various functional contexts, and hence their three-dimensional structures are subject to substantial structural constraints. For example, conservation of the β-tubulin axoneme motif (EGEF) across eukaryotic phyla, including *Tetrahymena*, is consistent with the hypothesis that assembly of a motile axoneme imposes structural restrictions that limit evolutionary divergence of its component β-tubulins [14], [15]. However, the presence of a single conserved motif does not preclude β-tubulin isotype function in other kinds of microtubules, as is true of BTU2. Different protein-protein interactions are likely to constrain the amino acid sequences of non-axonemal β-tubulins, such as the BLT isotypes. Thus, tubulins should be viewed as a conserved protein family that is subject to differential selective requirements that lead to subfunctionalization across the adaptive landscape [17]. In this context, we conclude that *Tetrahymena*, a single-celled organism with an exceptional diversity of microtubules [18], is an ideal model organism for analysis of the varied biological functions of divergent tubulin isotypes *in vivo*.

## Methods

### *Tetrahymena* strains and culture conditions

*T. thermophila* strains Cu 428.2, Mpr/Mpr [6-methylpurine-sensitive (6-mps65 ), VII], and Cu 427 ChxI-I/ChxI- [cycloheximide-sensitive (cy-s), VI] (generously provided by Drs. E. Hamilton and E. Orias, University of California, Santa Barbara) were used in this work. Unless specified otherwise, cells were grown in SPP medium (2% proteose peptone, 0.1% yeast extract, 0.2% glucose, 0.003% EDTA ferric sodium salt) at 30°C with moderate shaking. To prevent bacterial and fungal growth, the medium was supplemented with penicillin G (100 U/ml), streptomycin (100 μg/ml) and amphotericin B (0.025 μg/ml).

### RNA purification, synthesis of cDNA, and amplification of *Tetrahymena* β-tubulin cDNAs

Total RNA was extracted from 5-ml cultures of wild-type *T. thermophila* using the RNA Spin Mini RNA Isolation Kit (GE Healthcare, Milan, Italy). Treatment of total RNA with DNAse I was performed directly on the silica membrane. The quality of the RNA was examined by electrophoresis on a 1.2% agarose gel containing 2% formaldehyde. Potential contamination of the RNA by genomic DNA was tested by PCR amplification of 50 ng of total RNA using primers specific for *T. thermophila* 17S rDNA; the predicted fragment was not detected after 30 cycles, each consisting of 1 min denaturation at 94°C, 1 min primer annealing at 55°C, and 1 min elongation at 72°C.

First-strand cDNA was generated from 3 µg of total RNA using RevertAidM-MuLV Reverse Transcriptase (Fermentas, Milan, Italy) according to the manufacturer's directions. The cDNA was diluted 1∶5 with filtered, deionized water, and aliquots of l µl were used as template in PCR reactions designed to amplify the *BTU2*, *BLT1*, and *BLT4* genes:

1. *BTU2*: forward primer, ATGAGAGAAATCGTTCACATTCAAG; reverse primer, TCAGTTTTCACCTTCTTCTTCTTCGAA.
2. *BLT1*: forward primer, ATGAGAGAAATTGTTTGCATTTCAGCA; reverse primer, GCAATATTAGATTGTTATTTTGAGTTACATTTT.
3. *BLT4*: forward primer, ATGAGAGAAATTCTTAATATTCAAATAGG; reverse primer, TCAATTTTTATCTTTCTCTTCTCCAATTTC.

The primers were designed using sequence from the *T. thermophila* genome resource (TGD: http://www.ciliate.org). The PCR cycling parameters were identical to those specified in the preceding paragraph.

### DNA sequencing, sequence alignment, and three-dimensional modeling of BLTs

Sequencing of the *BLT* cDNAs and GFP-tagged tubulin reporter constructs (next section) was performed by BMRGENOMICS (Padua, Italy). Multiple sequence alignment of BLT1, BLT4, and BTU2 was performed using ClustalW 1.83 software [57]. The tertiary structures of *Tetrahymena* BLT1, BLT4, and BTU2 were obtained by comparative modeling to mammalian β-tubulin [27], [58] using MODELLER, version 9.1 (http://www.salilab.org/modeller/) [59].

### Generation of GFP-tagged tubulin reporter plasmids, transformation of *Tetrahymena* cells, and induction of the fusion proteins

The GFP-tagged tubulin reporter plasmids used in this work were engineered from pIGF-gtw (kindly provided by Dr. D. Chalker, Washington University, St. Louis, MO), which in turn was developed from the rDNA vector, pD5H8 [60], with several modifications. pIGF-gtw confers paromomycin resistance to transformed *Tetrahymena* cells through its mutated rDNA gene [61] and facilitates the cloning of coding sequences in frame with the GFP gene by inclusion of a Gateway recombination cassette (Invitrogen, Milan, Italy), a chloramphenicol-resistance gene, and a ccdB gene for negative selection [62]. Plasmids pIGF-BLT1, pIGF-BLT4, and pIGF-BTU2 were obtained by fusing the *GFP* gene in frame to the 5′ start codons of the *BLT1*, *BLT4* and *BTU2* coding sequences, respectively, using methods described by Cole et al. [63]. The resulting GFP-β-tubulin fusion proteins are under the transcriptional control of the CdCl_2_-inducible metallothionein promoter (MTT1).

pIGF-BLT and pIGF-BTU2 plasmids were introduced into *Tetrahymena* cells by conjugative electrotransformation of the complementary mating types Cu 427 and Cu 428.2 as previously described [64] and modified [65]. This selection protocol yields stable somatic (i.e., macronuclear) transformants that can be propagated indefinitely without conjugation. Briefly, complementary mating-type cells were starved in 10 mM Tris/HCl buffer (pH 7.5) for 16 h at 30°C. Equal numbers of complementary cells (3×10^5^/ml) were then mixed to initiate mating. After 9 h, cells were concentrated by centrifugation at 1600×g for 3 min, suspended in 10 mM Hepes buffer (pH 7.4) and electroporated with 20 μg of each plasmid as previously described [65]. After electroporation, cells were suspended in 5 ml of SPP plus antibiotics (penicillin G and streptomycin sulfate, 250 μg/ml each final concentration) followed by incubation at 30°C for 20 h before addition of the selective drug paromomycin sulfate (Sigma, Milan, Italy). The concentration of paromomycin sulfate was gradually increased from 100 to 900 µg/ml over a period of 7–8 days, and individual cells were selected to establish clonal lines. Five or more distinct clones obtained from each transformation were tested for the presence of the appropriate rDNA-GFP-β-tubulin constructs in exconjugant cells by PCR analysis; all were positive. The PCR products were sequenced to check that point mutations or other rearrangements did not occur during transformation.

Production of the GFP-tagged tubulins in transformed cell lines was induced by the addition of CdCl_2_ (final concentration 1 µg/ml) to the SPP medium. Two h after induction, cells were screened for GFP fluorescence by use of a Nikon Diaphot DMT inverted epifluorescence microscope equipped with an Apoplan 60× objective (1.4 NA). Cell lines whose fluorescence intensities were sufficiently strong to clearly delineate microtubule structures without high cytoplasmic backgrounds (clone 14 for pIGF-BLT4, clone 20 for pIGF-BLT4, clone 1 for pIGF-BTU2) were selected for further analysis. These clones were stored in 10 mM Tris-HCl (pH 7.5) at 20–24°C.

### Cell synchronization

*Tetrahymena* cells (2.5–7×10^4^ cells/ml) were synchronized by multiple heat shock treatments as previously described [66]. Each treatment entailed incubation at 42°C for 30 min followed by 30°C for 30 min. After the seventh heat shock, ∼80% of cells were found to be in mitosis when stained with 1% acetocarmine and examined by brightfield microscopy.

### Live imaging of the microtubule cytoskeleton in *Tetrahymena* cells expressing GFP-tagged β-tubulins

Somatic *Tetrahymena* cells, or conjugating pairs, were washed in 10 mM Tris-HCl (pH 7.5), transferred to 50% glycerol in 10 mM Tris-HCl (pH 7.5) to immobilize the cells, placed on a coverslip, and observed using the Nikon epifluorescence microscope system described above. Micrographs were recorded using a Nikon ND40 digital camera.

### Fixation of conjugating *Tetrahymena* cells for co-visualization of microtubules and nuclei

To visualize nuclei during conjugation, the cell pairs were fixed by the EtOH/Triton method [67]. Briefly, 1 ml of *Tetrahymena* cell pairs was harvested by centrifugation at 500 × g for 3 min. The pellet was resuspended in 0.5 ml of 10 mM Tris buffer (pH 7.5), and the cells were fixed in ice cold 50% ethanol, 0.1% Triton X-100 in PHEM buffer (60 mM PIPES, 25 mM HEPES, 10 mM EGTA, 2 mM MgCl_2_, final pH adjusted to 6.9 with NaOH). The cells were incubated at room temperature for 20 min, washed with PBS, then stained with propidium iodide (5 µg/ml ) for 5 min. After three washes with PBS, the cells were mounted over one drop of buffer containing 50% glycerol and the anti-fading agent propyl gallate. Micrographs were recorded using the Nikon microscope and digital camera described previously.

### Preparation of nuclei and cilia

Nuclei were purified from *T. thermophila* as previously described [68]. Cells were stirred in two volumes of hypotonic buffer [10 mM Tris (pH 7.5), 0.25 M sucrose, 10 mM MgCl_2_, 1 mM DTT, 3mM CaCl_2_] containing a cocktail of protease inhibitors (2 mM PMSF, 10 μg/ml pepstatin A, 10 μg/ml leupeptin, 1 μg/ml aprotinin, 0.25 μg/ml TAME) and 0.2% Triton X-100 for 5 min. To stop cell lysis, the sample was diluted by adding two volumes of the same buffer without Triton X-100 followed by centrifugation (500×g, 1 min, 4°C) to pellet unlysed cells and other cell debris. The supernatant was transferred into fresh tubes, and nuclei were recovered by centrifugation at 9,000×g for 2 min at 4°C. Nuclei were resuspended in hypotonic buffer and the purity checked by brightfield microscopy.

Cilia were purified as previously described [66]. Briefly, cells were starved in Tris-HCl (pH 7.4) for 16 h at 30°C, concentrated to 6×10^6^ cells/ml, and resuspended in four volumes of deciliating buffer [12% ethanol, 30 mM CaCl_2_, 1 mM EDTA, 2 mM MgSO_4_, 4 mM KCl, 2 mM 2-mercaptoethanol, 20 mM sodium acetate, 10 mM Tris-HCl (pH 7.0)]. Cells were incubated for 15 min on ice and then centrifuged at 1,100×g for 3 min. The pellet, composed of cell bodies, was checked by brightfield microscopy to determine the level of deciliation, which typically was ∼90%. To recover cilia, the supernatant was centrifuged at 14,000×g for 20 min.

### SDS-polyacrylamide gel electrophoresis (PAGE) and immunoblotting

SDS-PAGE was performed by the method of Laemmli [69]. After electrophoresis, gels (9% polyacrylamide) were blotted electrophoretically to nitrocellulose sheets (0.45 μm, GE Healthcare, Milan, Italy) using standard conditions [70]. Blots were developed using a rabbit polyclonal anti-GFP primary antibody (1∶1,000 dilution; Sigma, Milan, Italy) and peroxidase-conjugated goat anti-rabbit IgG secondary antibody (1∶1,000 dilution; GE Healthcare, Milan, Italy). Bound secondary antibody was detected by Enzyme-Coupled Luminescence (ECL^TM^, GE Healthcare, Milan, Italy).

### Chemicals, materials, and reagents

*Taq* polymerase and DNA modifying and restriction enzymes were purchased from Fermentas (Milan, Italy). Oligonucleotides were synthesized by Sigma/Genosys (Milan, Italy). All routine chemicals were of analytical grade and supplied by Sigma Aldrich (Milan, Italy).

## Supporting Information

Table S1 **Amino acid compositions of BLT1, BLT4, and BTU2.**
(DOC)
