# Supplementary material for: Distinct Functional Roles of β-Tubulin Isotypes in Microtubule Arrays of Tetrahymena thermophila, a Model Single-Celled Organism
Source: PLoS One. 2012 Jun 22;7(6):e39694. doi: 10.1371/journal.pone.0039694 (PMC3382179; doi:10.1371/journal.pone.0039694)
Supplement: Table S1 — Amino acid compositions of BLT1, BLT4, and BTU2. (DOC) [file pone.0039694.s001.doc]

**Table** S1. Amino acid compositions of BLT1, BLT4, and BTU2

| Amino acid/Amino acid category | BLT1  (%) | BLT4  (%) | BTU2  (%) |
| --- | --- | --- | --- |
| Ala | 5.5 | 5.0 | 6.7 |
| Cys | 1.9 | 1.8 | 2.1 |
| Asp | 4.9 | 5.5 | 5.5 |
| Glu | 7.9 | 7.9 | 8.7 |
| Phe | 4.7 | 5.0 | 5.3 |
| Gly | 6.1 | 5.5 | 8.7 |
| His | 2.5 | 1.3 | 2.3 |
| Ile | 5.9 | 10.1 | 4.6 |
| Lys | 4.2 | 5.7 | 3.5 |
| Leu | 7.2 | 8.8 | 7.6 |
| Met | 3.2 | 2.2 | 4.4 |
| Asn | 6.8 | 7.2 | 5.1 |
| Pro | 3.2 | 4.8 | 4.4 |
| Gln | 7.0 | 5.5 | 3.0 |
| Arg | 4.7 | 3.5 | 5.1 |
| Ser | 9.1 | 7.0 | 5.8 |
| Thr | 3.4 | 4.4 | 6.4 |
| Val | 6.8 | 3.3 | 6.7 |
| Trp | 0.8 | 0.7 | 0.9 |
| Tyr | 4.0 | 5.0 | 3.5 |
| Aromatic^a^ | 9.5 | 10.7 | 9.7 |
| Aliphatic^b^ | 18.8 | 22.0 | 18.9 |
| Charged^c^ | 11.5 | 23.8 | 25.1 |
| Polar (uncharged)^d^ | 25.0 | 30.8 | 25.8 |
| Hydrophobic^e^ | 39.5 | 40.0 | 39.6 |
| Small^f^ | 51.3 | 44.4 | 51.3 |

^a^ Determined by summing the percentages of Phe, Trp and Tyr.

^b^ Determined by summing the percentages of Ile, Leu and Val.

^c^ Determined by summing the percentages of Asp, Glu, His, Lys and Arg.

^d^ Determined by summing the percentages of Cys, Ser, Thr, Asn, Gln, and Tyr.

^e^ Determined by summing the percentages of Ile, Leu, Val, Phe, Trp, Tyr, Ala, and Met.

^f^ Determined by summing the percentages of Gly, Cys, Pro, Ala, Val, Ser, Thr, Asn and Asp.
